# An Experimental Study of a Zeolite Membrane Reactor for Reverse Water Gas Shift

**DOI:** 10.3390/membranes12121272

**Published:** 2022-12-15

**Authors:** Motomu Sakai, Kyoka Tanaka, Masahiko Matsukata

**Affiliations:** 1 Research Organization for Nano & Life Innovation, Waseda University, 513 Wasedatsurumaki-cho, Shinjuku-ku, Tokyo 162-0041, Japan; 2 Department of Applied Chemistry, Waseda University, 513 Wasedatsurumaki-cho, Shinjuku-ku, Tokyo 162-0041, Japan; 3 Advanced Research Institute for Science and Engineering, Waseda University, 513 Wasedatsurumaki-cho, Shinjuku, Tokyo 162-0041, Japan

**Keywords:** zeolite, reverse water gas shift, membrane reactor, carbon recycle, MFI, carbon dioxide, hydrogen, dehydration, water

## Abstract

Reverse water gas shift (RWGS) is attracting attention as one of the promising technologies for CO_2_ conversion. Selective removal of H_2_O from the reaction system can improve the CO_2_ conversion beyond the equilibrium conversion of RWGS in a conventional reactor. In this study, a conventional plug-flow reactor without membrane, and two types of RWGS membrane reactors using ZSM-5 membranes, were developed. The yield of CO without membrane (*Case 1*) was almost the same as the equilibrium conversion. A membrane reactor (*Case 2*) showed a CO yield 2–3% above that of a conventional reactor. From the results, the effectiveness of the dehydration membrane reactor for RWGS was verified. In addition, CO yield was further increased in the reactor made up of the combination of conventional reactor and membrane reactor (*Case 3*). For example, the CO yields in *Cases 1*, *2*, and *3* at 560 K were 21.8, 24.9, and 29.0%, respectively. Although the CO yield increased in *Case 2*, a large amount of raw materials penetrated through the membrane to the permeation side, and was lost. In *Case 3*, H_2_ and CO_2_ permeation through the membrane were suppressed because of the existence of H_2_O, resulting in the prevention of the leakage of raw material, and contributing to the high CO yield.

## 1. Introduction

Technologies to reduce the emission of greenhouse gas (GHG) have widely been studied and implemented. Among GHG emission, the reduction of CO_2_ emission is still a key challenge. There are three approaches for CO_2_ emission reduction; suppression of CO_2_ production, CO_2_ storage (CCS) and CO_2_ utilization (CCU). In particular, CO_2_ is no longer considered a waste, but a raw material for chemicals and fuels produced with the addition of renewable energy and H_2_ in CCU [1,2,3].

Reverse water gas shift (RWGS) is attracting attention as one of the promising techniques for expanding CCU. CO produced by RWGS can be converted to chemicals and fuels through so-far developed processes such as methanol synthesis and Fischer–Tropsch synthesis.

For efficient utilization of CO_2_, high conversion is required in RWGS. However, the conversion of CO_2_ in RWGS is strongly limited by thermodynamic equilibrium. RWGS is an endothermic reaction; thus, a high reaction temperature is required to achieve high conversion. For example, CO_2_ conversion of 75% is finally achieved at 1073 K in the case of the feed composition of CO_2_:H_2_ = 1:3. However, such a high reaction temperature often causes the deterioration of both the catalyst and the reactor. When lowering the reaction temperature is accomplished by using a membrane reactor, not only is the lifetime of catalyst and equipment extended, but the reduction of energy consumption is expected.

Selective removal of H_2_O from the reaction system can improve CO_2_ conversion in RWGS. Although the potential of membranes used for in situ H_2_O removal from RWGS reactor was pointed out, a very little research about membrane reactors equipped with dehydration membranes has been reported [1,4,5]. Lee et al. reported an RWGS membrane reactor with a polyimide hollow fiber membrane for dehydration. In this study, the hollow fiber membrane reactor delivered a three-fold increase in the CO yield at 523 K, due to selective H_2_O removal [4]. Although the polyimide membrane exhibited great permselectivity for H_2_O, it was not suitable for high temperatures above 573 K because of its thermal stability.

Here, we suggest a membrane reactor for RWGS using zeolite membrane. Some zeolite membranes exhibit good H_2_O selectivity even at high temperatures [6,7,8,9]. Therefore, zeolite membranes have been studied for dehydration membrane reactors, and especially for methanol synthesis [7,8,9]. A hydrophilic ZSM-5 membrane with selectivity for water vapor was developed in our previous study [10]. In this study, an RWGS membrane reactor equipped with a ZSM-5 membrane was developed. The effect of the configuration of membrane reactor on the CO yield was investigated.

## 2. Experimental

### 2.1. Catalyst Preparation

CuO/ZnO/γ-Al_2_O_3_ was used as a catalyst in RWGS. CuO/ZnO/γ-Al_2_O_3_ was prepared by a polymerized complex method with reference to a previous report [11]. Cu (NO_3_)_2_·3H_2_O (FUJIFILM Wako Chemical, Osaka, Japan), and Zn (NO_3_)_2_·6H_2_O (FUJIFILM Wako Chemical, Osaka, Japan) were used as metal sources. 3.36 g of Cu (NO_3_)_2_·3H_2_O, 4.14 g of Zn (NO_3_)_2_·6H_2_O and 0.560 g of γ-Al_2_O_3_ were dissolved in 100 g of distilled water. 100 mL distilled water with 16.0 g citric acid (FUJIFILM Wako Chemical, Osaka, Japan) dissolved was added to the solution. After adding 5.18 g of ethylene glycol (FUJIFILM Wako Chemical, Osaka, Japan), the solution was heated at 423 K for 1 h under stirring conditions. Finally, produced powder was calcined at 623 K for 4 h, and then CuO/ZnO/γ-Al_2_O_3_ was obtained.

H_2_ reduction was carried out for the catalyst prior to use in the reaction, at 623 K for 1 h in H_2_ stream.

### 2.2. Membrane Preparation

The ZSM-5 membrane was synthesized following the previous report [12]. The ZSM-5 membrane was prepared on the outer surface of porous support by a secondary growth method. A porous tubular α-Al_2_O_3_ with an outer diameter of 10 mm, an inner diameter of 7 mm and a length of 90 mm was used as support. The effective membrane area was 25.1 cm^2^. ZSM-5 seed crystal was loaded on a support using a dip-coating method. Synthesis solution with the molar composition of Al_2_O_3_:240SiO_2_:53.3Na_2_O:8000H_2_O was prepared by mixing sodium aluminate (Na_2_O, 33 wt%; Al_2_O_3_, 37 wt%; Kanto Chemical Co., Tokyo, Japan), colloidal silica (ST-S, Nissan Chemical Ind. Ltd., Yokohama, Japan), sodium hydroxide (Kanto Chemical, Tokyo, Japan) and distilled water. A seeded support was immersed in a synthesis solution and hydrothermally treated at 453 K for 16 h in a PTFE-lined autoclave. After hydrothermal treatment, the autoclave was quenched by flowing tap water. The membrane was rinsed with boiling water and dried at 383 K over 10 h. The details of the synthesis procedure are shown elsewhere [12].

### 2.3. Membrane Reactor Test

**Figure 1:**
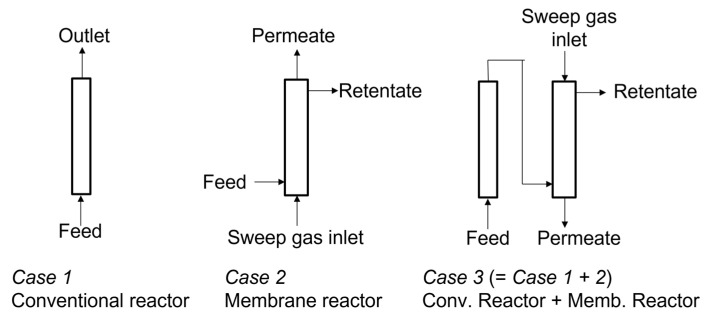
Configurations of RWGS reactors. *Case 1*, conventional plug-flow reactor; *Case 2*, membrane reactor; *Case 3*, combination of conventional and membrane reactors.

Three types of reactors were used for RWGS. One of them was a conventional plug-flow reactor without a membrane (*Case 1*). The second one was a plug-flow reactor with a ZSM-5 membrane (*Case 2*). The third one is the combination of a conventional reactor and membrane reactor; in particular; a conventional reactor was located before a membrane reactor (*Case 3*). Figure 1 shows the configurations of RWGS reactors.

**Figure 2:**
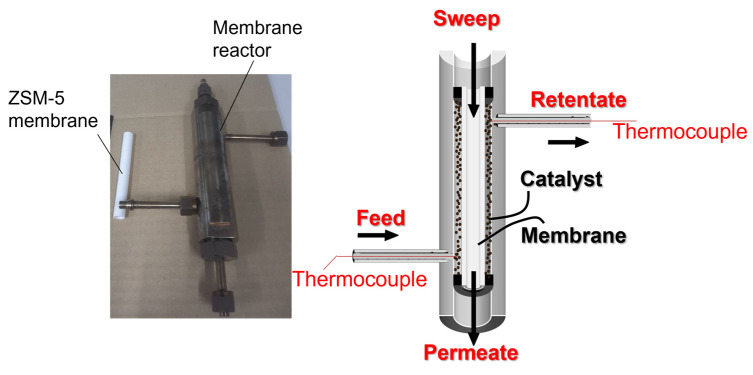
Picture and schematic diagram of membrane reactor.

In the membrane reactors, a catalyst was placed around the outer surface of tubular membrane. 0.8 g and 3.2 g of catalysts were loaded in the conventional reactor in *Case 1* and the membrane reactor in *Case 2*, respectively. In *Case 3*, 4.0 g of catalyst was used in total. The temperature of the catalyst bed was evaluated by a thermocouple, inserted directly. Figure 2 shows a picture and schematic diagram of the membrane reactor. The length and inner diameter of the membrane reactor were 120 and 12 mm, respectively.

A tubular membrane was fixed in the membrane reactor by graphite O-ring. The mixture of CO_2_ and H_2_ (CO_2_/H_2_ = 1/3) was fed to the outer surface of the membrane. Permeate was swept with flowing Ar. The feed, retentate and permeate gas were analyzed by GC-TCD (GC-8A, Shimadzu Corp., Kyoto, Japan) for their compositions. Material balance, *B*, and CO yield, *Y_CO_*, were determined by following equations:

(1) $$B\;[\%]=\frac{F_{{CO}_{2}\;perm.+reten.\;}\left[{\mathrm{mol}\;s}^{-1}\right]+F_{CO\;perm.+reten.\;}\left[{\mathrm{mol}\;s}^{-1}\right]}{F_{{CO}_{2}\;feed}\left[{\mathrm{mol}\;s}^{-1}\right]\;}\;\times \;100$$

(2) $$Y_{\mathrm{CO}}\;[\%]=\frac{F_{CO\;perm.+reten.\;}\left[{\mathrm{mol}\;s}^{-1}\right]}{F_{{CO}_{2}\;feed}\left[{\mathrm{mol}\;s}^{-1}\right]\;}\;\times \;100$$

where *F_i feed_* was the flow rate of component *i* in feed stream. *F_i perm.+reten._* corresponded to the sum of the component *i* flow rate in permeate and retentate. In *Case 1*, *F_CO perm.+reten._* shows the flow rate at the outlet.

## 3. Results and Discussion

### 3.1. Characterizations of Catalyst and Membrane

**Figure 3:**
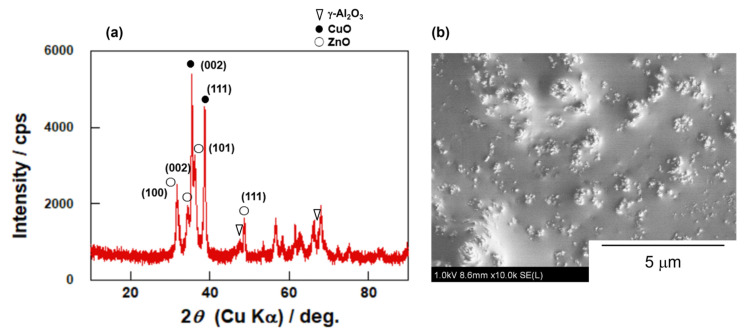
(**a**) XRD patterns and (**b**) typical FE-SEM images of CuO/ZnO/γ-Al_2_O_3_ catalyst.

Figure 3 shows the XRD pattern and typical FE-SEM images of the CuO/ZnO/γ-Al_2_O_3_ catalyst. The peaks corresponding to CuO, ZnO and γ-Al_2_O_3_ were observed using XRD. The catalyst was observed to be aggregates of particles of hundreds of nm. The composition of CuO/ZnO/Al_2_O_3_ was detected as 41/39/20 wt% by ICP, which agreed with the feed composition in preparation, as described in the experimental section.

Although as-made, the CuO/ZnO/γ-Al_2_O_3_ catalyst was powder, the catalyst was granulated for use in the membrane reactor test. By pressurization with 60 kN for 15 min, a granular catalyst with a diameter of 0.25–0.50 mm was obtained. The photographs of catalyst before and after granulation are shown in Figure S1 in the Supplementary Materials.

**Figure 4:**
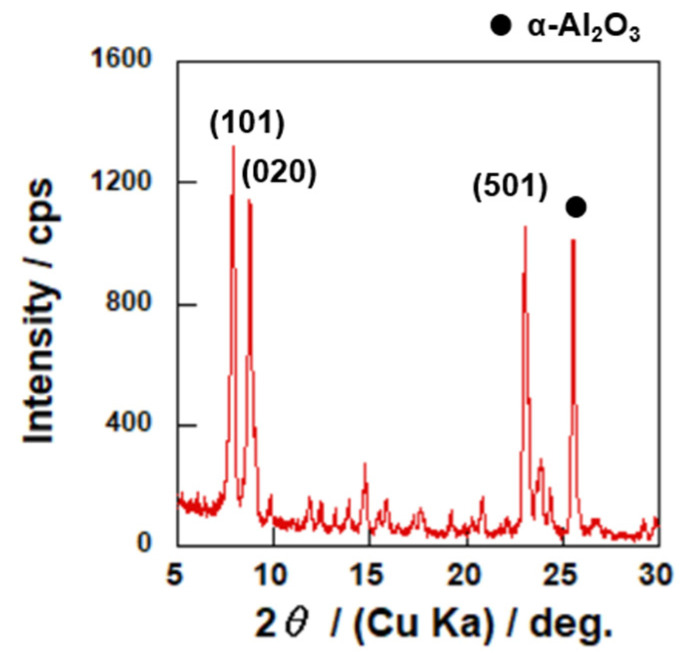
XRD pattern of prepared membrane.

**Figure 5:**
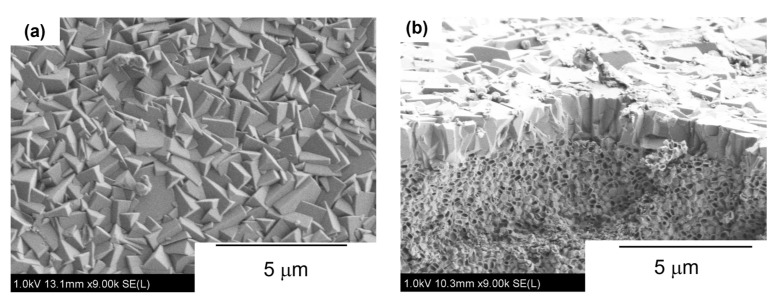
Typical FE-SEM images of prepared membrane. (**a**) surface; (**b**) cross-section.

Figure 4 and Figure 5 show the XRD pattern and typical FE-SEM images of the prepared membrane. Figure 4 shows no apparent reflection peaks other than those corresponding to the MFI-type zeolite and α-alumina used as support. The result from the microscopic analysis revealed that the support surface was fully covered with compact and well inter-grown crystals. The Si/Al ratio of the continuous layer on the outer surface was ca. 18, measured by EDS. As a result, the obtained membrane was mainly composed of ZSM-5 crystals having the MFI-type structure.

The results shown in Figure 4 and Figure 5 suggested that a pure ZSM-5 membrane layer was synthesized on the outer surface of the α-Al_2_O_3_ support by a secondary growth method.

### 3.2. Permeation and Separation Properties of MFI Zeolite for H_2_O/H_2_ and H_2_O/CO_2_

Separation tests for the binary mixtures of H_2_O/H_2_ and H_2_O/CO_2_ were performed to evaluate the potential of the ZSM-5 membrane for reverse water gas shift. A separation test was carried out in the temperature range of 523–623 K for the equimolar mixtures of H_2_O/H_2_ and H_2_O/CO_2_. To study the separation principle of the ZSM-5 membrane, permeation tests for single components of H_2_O, H_2_ and CO_2_ were conducted as well.

Table 1 shows the results of separation tests for binary mixtures and single gas permeation tests. The partial pressures of H_2_O and H_2_ or CO_2_ were adjusted at 10 and 90 kPa in binary systems. In unary systems, the partial pressure of each component was 100 kPa. The ZSM-5 membrane showed H_2_O selectivity for both the H_2_O/H_2_ and H_2_O/CO_2_ mixture. In H_2_O/H_2_ separation, H_2_O permeance was 2.99 × 10^−7^ mol m^−2^ s^−1^ Pa^−1^ with a separation factor of 17.2 at 523 K. The permeance and separation factor through the ZSM-5 membrane for the H_2_O/CO_2_ mixture were 3.64 × 10^−7^ mol m^−2^ s^−1^ Pa^−1^ and 17.8, respectively. In both systems, separation factors tended to be higher at lower membrane temperatures; e.g., the H_2_O separation factor for H_2_O/CO_2_ slightly decreased to 11.6 with increasing temperature up to 623 K. In single gas permeation, H_2_ and CO_2_ permeances were an order of magnitude larger than those in binary systems with H_2_O. In other words, H_2_ and CO_2_ permeation through the ZSM-5 membrane was strongly hindered by the existence of H_2_O.

Here, the principle of H_2_O selectivity through the ZSM-5 membrane was discussed. H_2_O would preferentially adsorb in the micropore of ZSM-5 because of the high relative pressure of H_2_O in gas phase and the strong affinity between H_2_O and hydrophilic zeolite. Consequently, the permeations of the other gases were blocked by adsorbed H_2_O. Sawamura et al. reported that the ZSM-5 membrane showed both H_2_O selectivity for a H_2_O/H_2_ mixture and methanol selectivity for a methanol/H_2_ mixture, similarly [10]. In addition, it is known that such affinity-based separation occurred in propylene/propane separation through Ag^+^-zeolite membrane and toluene/CH_4_ separation through an ionic liquid-containing silica membrane [13,14,15,16]. In principle, the adsorption amount of H_2_O in micropore decreased at higher temperatures, resulting in less blocking for gas permeation and lower separation performance.

From the result of the separation tests listed in Table 1, the good H_2_O selectivity of ZSM-5 membrane, even at a high temperature of 623 K where the reactor of RWGS is operated, was verified. 

### 3.3. RWGS Membrane Reactor Tests

A conventional plug-flow reactor without a membrane and two types of RWGS membrane reactors with ZSM-5 membranes were developed, as described in the experimental section. *Case 1* was a conventional reactor that was composed of a plug-flow reactor. *Case 2* was a flow-type membrane reactor with a tubular ZSM-5 membrane surrounded by a catalyst. *Case 3* was made up of the combination of *Case 1* and *Case 2*; e.g., a membrane reactor was located posteriorly to a conventional reactor. The reaction temperature was controlled in the range of 515–609 K. The component of feed gas was adjusted as CO_2_:H_2_ = 1:3.

**Figure 6:**
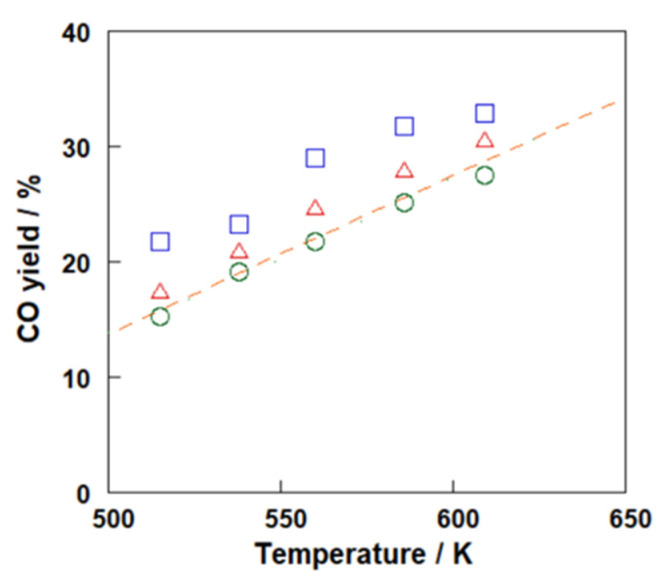
Comparison of CO yields obtained in ◦, *Case 1*; ▵, *Case 2* and □, *Case 3*. The dashed line shows the equilibrium yield.

Figure 6 shows CO yield through each reactor. In addition, Table 2 lists the material balance of each condition. It is noted that the material balance in all conditions was 98.1–103.4%, suggesting that the analyses in these experiments were very accurate, and thus the data were sufficient to discuss the differences in the reactor configuration. Unsurprisingly, the yield of CO without membrane, in *Case 1*, was almost the same as the equilibrium limitation. This result meant that the reaction rate was large enough under the conditions. In *Case 2*, a membrane reactor showed a CO yield of 2–3% above that of *Case 1* and the equilibrium. Moreover, the yields of CO in *Case 3* were even greater than those in *Case 2*. For example, the CO yields in *Cases 1*, *2,* and *3* at 560 K were 21.8, 24.9, and 29.0%, respectively.

**Figure 7:**
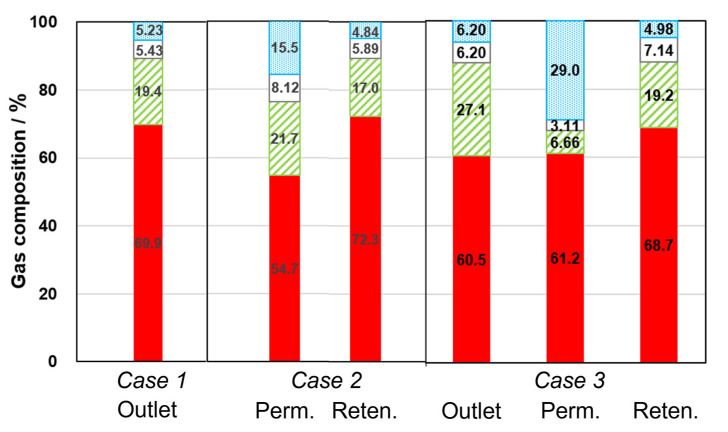
Exit gas compositions of each reactor at 560 K. The bars from bottom to top showed H_2_, CO_2_, CO and H_2_O.

Figure 7 shows the exit gas compositions of each reactor at 560 K. Depending on the number of exits in the reactor, the number of bars in Figure 7 is different. In particular, while the conventional reactor (*Case 1*) had only one outlet, the membrane reactor (*Case 2*) had two exits, permeate and retentate. In *Case 3*, the gas compositions at the three exits were shown, the outlet of conventional reactor (feed gas for the membrane reactor), permeate and retentate. In the Supplementary Materials, gas compositions at different temperatures are shown in Figure S2.

In *Case 1*, the gas composition had a good agreement with the equilibrium composition because the conversion of CO_2_ reached the ceiling of thermodynamic equilibrium. Thus, the content of H_2_O and CO were both ca. 5%. In this case, *K* calculated by the following equation was 0.0202, almost the same as the equilibrium constant, *K*_560_ = 0.0205 [17].

(3) $$K=\frac{\left[\mathrm{CO}\right]\left[H_{2}O\right]}{\left[{\mathrm{CO}}_{2}\right]\left[H_{2}\right]\;}$$

In *Case 2*, although the retentate composition was almost the same as in *Case 1*, the ratio of H_2_O was slightly small because of selective dehydration by the ZSM-5 membrane. In contrast, the H_2_O content in permeate showed a relatively large value of 15.5%. Here, *K,* calculated from the sum of permeate and retentate, was 0.0385, which exceeded *K*_560_. This result suggested that the ZSM-5 membrane maintained good H_2_O separation performance in the RWGS membrane reactor and the equilibrium shifted according to *Le Chatelier*’s law. In addition, the H_2_O content in permeate further increased up to 29.0% in *Case 3*. *K,* calculated from the sum of permeate and retentate, also increased to 0.0460, which overwhelmed both *Cases 1* and *2*. The ratios of H_2_ and CO_2_ in permeate in *Case 3* were suppressed compared with *Case 2*, which investigated that the membrane showed high a separation performance of H_2_O in *Case 3*.

It is noted that the relationship of gas compositions among *Cases 1* to *3* was almost the same at all temperatures (all data is shown in Figure S2). The equilibria were shifted in *Cases 2* and *3* by selective removal of H_2_O. The H_2_O content in permeates in *Case 3* was always larger than those in *Case 2* in the temperature range of 515–609 K. In addition, the time course of the CO yield in *Case 3* is shown in Figure S3. The CO yield was stable at each temperature, suggesting that the deteriorations of catalyst and membrane did not occur, at least during the test’s 12 h.

Here, the effect of the reactor configuration on membrane property and CO yield was discussed. The ZSM-5 membrane had high H_2_ and CO_2_ permeance under dry conditions as described in Table 1. In *Case 2*, the extent of reaction was low near the entrance of the reactor, and the partial pressure of H_2_O was also small. Therefore, H_2_ and CO_2_ would easily penetrate through the ZSM-5 membrane near the entrance of reactor, resulting in a decrease in partial pressures of feed components. On the other hand, the relatively higher partial pressure of H_2_O was kept at the entrance of the membrane reactor, because the reaction almost achieved equilibrium in the conventional reactor located before the membrane reactor in *Case 3*. Consequently, H_2_ and CO_2_ permeation were suppressed in the membrane reactor, resulting that the prevention of the leakage of raw material that contributed to the high CO yield in *Case 3*.

Finally, CO yield was successfully increased by the membrane reactor equipped with a ZSM-5 membrane. The yield at 560 K in *Case 3* corresponds to the thermodynamic equilibrium conversion at 616 K. In other words, the reaction temperature could be lowered by 56 K by using a reactor of a combination of a conventional reactor and membrane reactor.

## 4. Conclusions

Three types of reactors for RWGS were developed, and the effect of each configuration on CO yield was investigated.

The yield of CO without a membrane *(Case 1*) was almost the same as the equilibrium conversion. A membrane reactor (*Case 2*) exhibited a CO yield 2–3% above that of a conventional reactor. In addition, CO yield was further increased by the reactor made up ofcombination of a conventional reactor and a membrane reactor (*Case 3*). The yields of CO in *Cases 1, 2,* and *3* at 560 K were 21.8, 24.9, and 29.0%, respectively. From the results, the effectiveness of a dehydration membrane reactor for RWGS was verified.

CO yield was improved by introducing a conventional reactor before the membrane reactor in *Case 3*. The prevention of leakage of raw materials from the feed side to the permeation side would cause such a promising result. H_2_ and CO_2_ easily penetrated through the ZSM-5 membrane because of the absence of H_2_O near the entrance of the membrane reactor in *Case 2*. In contrast, the permeations of raw materials in the membrane reactor were blocked by H_2_O, because H_2_O was generated in the conventional reactor before the membrane reactor in *Case 3*.

The CO yield of 29.0% at 560 K in *Case 3* corresponds to the thermodynamic equilibrium conversion at 616 K. In other words, the reaction temperature could be lowered by 56 K by using a reactor of a combination of a conventional reactor and a membrane reactor. Such an advantageous effect of the membrane reactor would further be improved by the improvement of membrane performance.

## Figures and Tables

**Table 1:** Results of separation tests for binary mixtures and single gas permeation tests.

System	Temperature/K	Permeance/10^−7^ mol m^−2^ s^−1^ Pa^−1^			Separation Factor/-
		H_2_O	H_2_	CO_2_	
H_2_O	523	3.89	-	-	
	623	3.58	-	-	
H_2_	523	-	1.73	-	
	623	-	2.68	-	
CO_2_	523	-	-	1.31	
	623	-	-	1.60	
H_2_O/H_2_	523	2.99	0.154	-	17.2
	623	1.78	0.204	-	8.11
H_2_O/CO_2_	523	3.64	-	0.182	17.8
	623	2.24	-	0.180	11.6

**Table 2:** Carbon balances in each reactor.

Configuration	Carbon Balance/%				
	515 K	538 K	560 K	586 K	609 K
*Case 1*	101.0	100.6	99.8	99.8	99.7
*Case 2*	100.7	100.4	103.4	100.1	102.1
*Case 3*	98.1	99.6	99.4	98.0	99.0

## Data Availability

Not applicable.

## Supplementary Materials

The following supporting information can be downloaded at: https://www.mdpi.com/article/10.3390/membranes12121272/s1, Figure S1: The photographs of catalysts; Figure S2: The outlet gas compositions of each reactor at 515, 538, 586, and 609 K. Figure S3: The time course of CO yield in *Case 3*.
