# Supplementary material for: An Experimental Study of a Zeolite Membrane Reactor for Reverse Water Gas Shift
Source: Membranes (Basel). 2022 Dec 15;12(12):1272. doi: 10.3390/membranes12121272 (PMC9784740; doi:10.3390/membranes12121272)
Supplement: Supplementary file 1 [file membranes-12-01272-s001.zip › membranes-2099662-supplementary.pdf]

Supplementary Materials

Title: An experimental study of zeolite membrane reactor for reverse water gas shift

Authors: Motomu Sakai[1*], Kyoka Tanaka[2] and Masahiko Matsukata[1,2,3]

Affiliation:

1 Research Organization for Nano & Life Innovation, Waseda University, 513 Wasedatsurumaki-cho, Shinjuku-ku, Tokyo 162-0041, Japan

2 Department of Applied Chemistry, Waseda University, 513 Wasedatsurumaki-cho, Shinjuku-ku, Tokyo 162-0041, Japan

3 Advanced Research Institute for Science and Engineering, Waseda University, 513 Wasedatsurumaki-cho, Shinjuku-ku, Tokyo 162-0041, Japan

***Corresponding author:**

Phone number: +81-3-5286-3850

e-mail address: saka.moto@aoni.waseda.jp

Postal address: 513 Wasedatsurumaki-cho, Shinjuku-ku, Tokyo 162-0041, Japan

Figure S1 shows the photographs of catalysts.

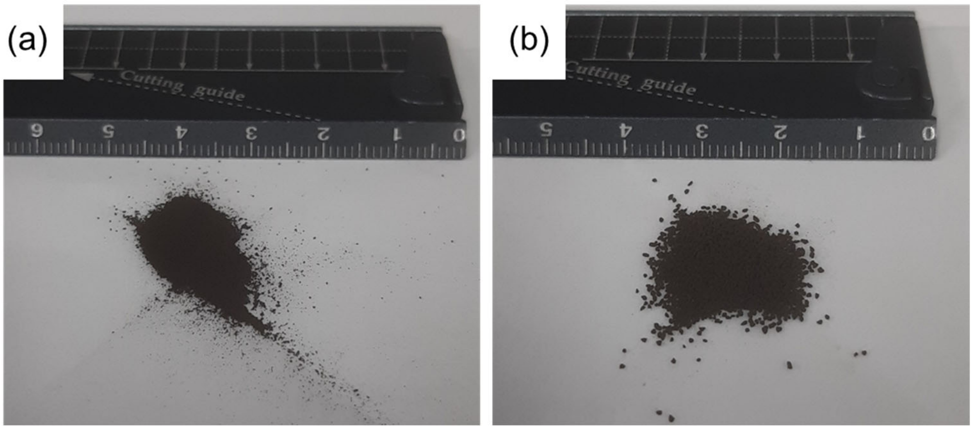

Figure S1 The photographs of catalysts. (a) as-made; (b) after granulation

Figure S2 shows the exit gas compositions of each reactor.

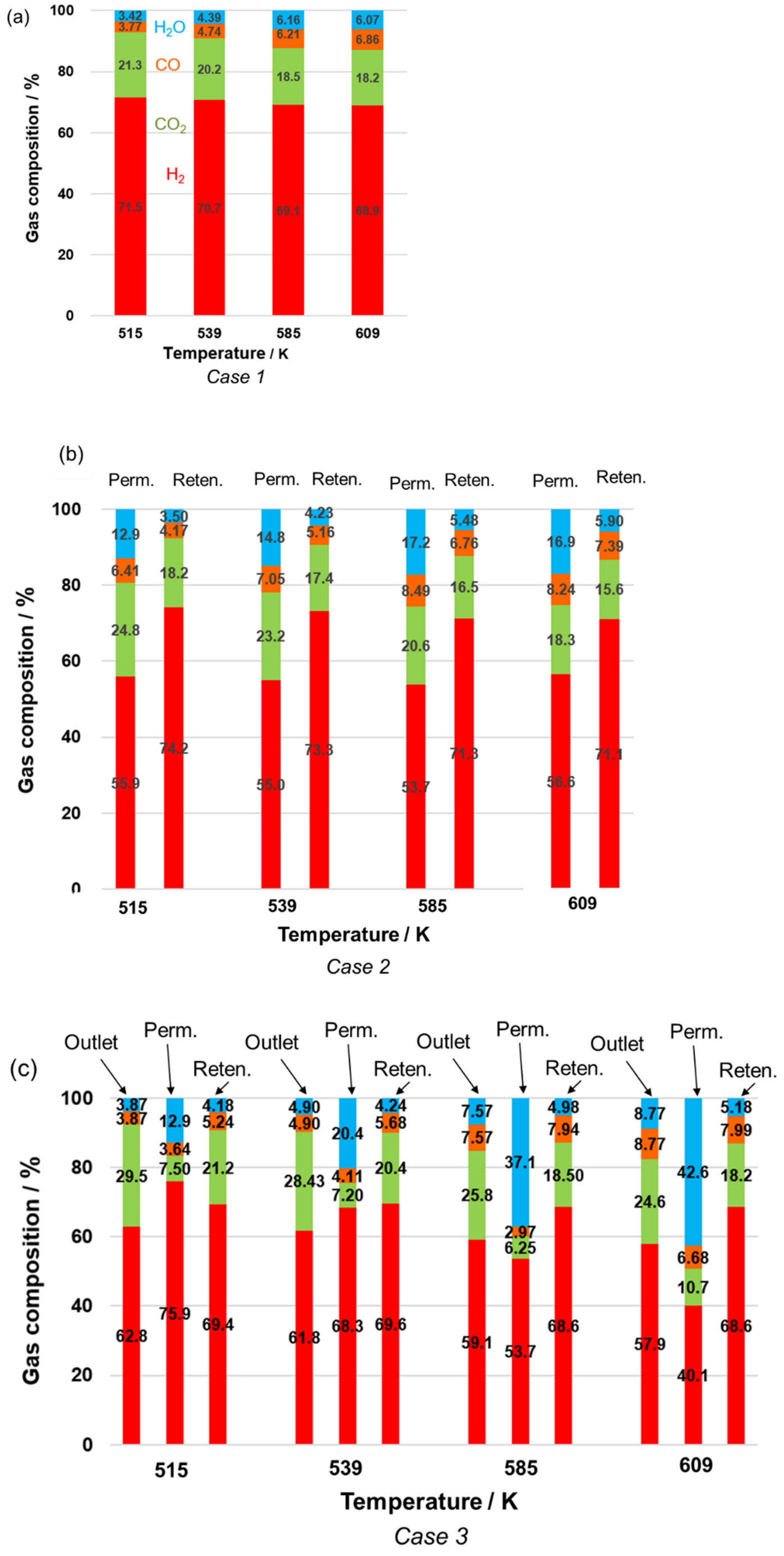

Figure S2 The exit gas compositions of each reactor. (a) *Case 1*; (b) *Case 2*; (c) *Case 3*.

Fig. S3 shows the time course of CO yield in *Case 3*. The CO yield was stable at each temperature at least during the test, 12 h.

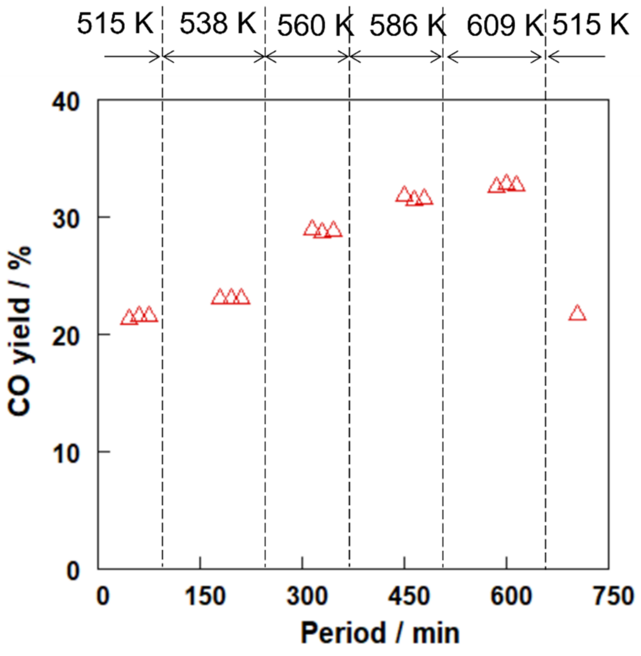

Figure S3 The time course of CO yield in *Case 3*.
